# Sensory Systems and Transcriptional Regulation in *Escherichia coli*

**DOI:** 10.3389/fbioe.2022.823240

**Published:** 2022-02-14

**Authors:** Georgette Femerling, Socorro Gama-Castro, Paloma Lara, Daniela Ledezma-Tejeida, Víctor H. Tierrafría, Luis Muñiz-Rascado, César Bonavides-Martínez, Julio Collado-Vides

**Affiliations:** ^1^ Centro de Ciencias Genómicas, Universidad Nacional Autónoma de México, Cuernavaca, México; ^2^ Institute of Molecular Systems Biology, ETH Zürich, Zurich, Switzerland; ^3^ Department of Biomedical Engineering, Boston University, Boston, MA, United States; ^4^ Centre for Genomic Regulation (CRG), The Barcelona Institute of Science and Technology, Universitat Pompeu Fabra (UPF), Barcelona, Spain

**Keywords:** signal, gene regulation, sensory-response genetic units, feedback, biosensor, transcriptional regulatory network, *E. coli*, transcription factor

## Abstract

In free-living bacteria, the ability to regulate gene expression is at the core of adapting and interacting with the environment. For these systems to have a logic, a signal must trigger a genetic change that helps the cell to deal with what implies its presence in the environment; briefly, the response is expected to include a feedback to the signal. Thus, it makes sense to think of genetic sensory mechanisms of gene regulation. *Escherichia coli* K-12 is the bacterium model for which the largest number of regulatory systems and its sensing capabilities have been studied in detail at the molecular level. In this special issue focused on biomolecular sensing systems, we offer an overview of the transcriptional regulatory corpus of knowledge for *E. coli* that has been gathered in our database, RegulonDB, from the perspective of sensing regulatory systems. Thus, we start with the beginning of the information flux, which is the signal’s chemical or physical elements detected by the cell as changes in the environment; these signals are internally transduced to transcription factors and alter their conformation. Signals transduced to effectors bind allosterically to transcription factors, and this defines the dominant sensing mechanism in *E. coli*. We offer an updated list of the repertoire of known allosteric effectors, as well as a list of the currently known different mechanisms of this sensing capability. Our previous definition of elementary genetic sensory-response units, GENSOR units for short, that integrate signals, transport, gene regulation, and the biochemical response of the regulated gene products of a given transcriptional factor fit perfectly with the purpose of this overview. We summarize the functional heterogeneity of their response, based on our updated collection of GENSORs, and we use them to identify the expected feedback as part of their response. Finally, we address the question of multiple sensing in the regulatory network of *E. coli*. This overview introduces the architecture of sensing and regulation of native components in *E.coli* K-12, which might be a source of inspiration to bioengineering applications.

## Introduction

The ability to adapt to changes in the environment is a fundamental property of life, which in bacterial systems has been studied for decades at the molecular level, thanks to advances in genetics and the relative simplicity of these organisms.

During more than two and a half decades our laboratory has been gathering knowledge on the regulation of transcription initiation and the organization and expression of the regulated genes in *Escherichia coli* K-12. This knowledge can be accessed in two databases, RegulonDB, and EcoCyc (Santos-Zavaleta et al., 2019; Keseler et al., 2021). This corpus shows the complex architecture of multiple sensing and regulatory systems currently known in *E. coli* and is the basis for the review presented here from the perspective of sensing systems.

### RegulonDB Overview

**FIGURE 1:**
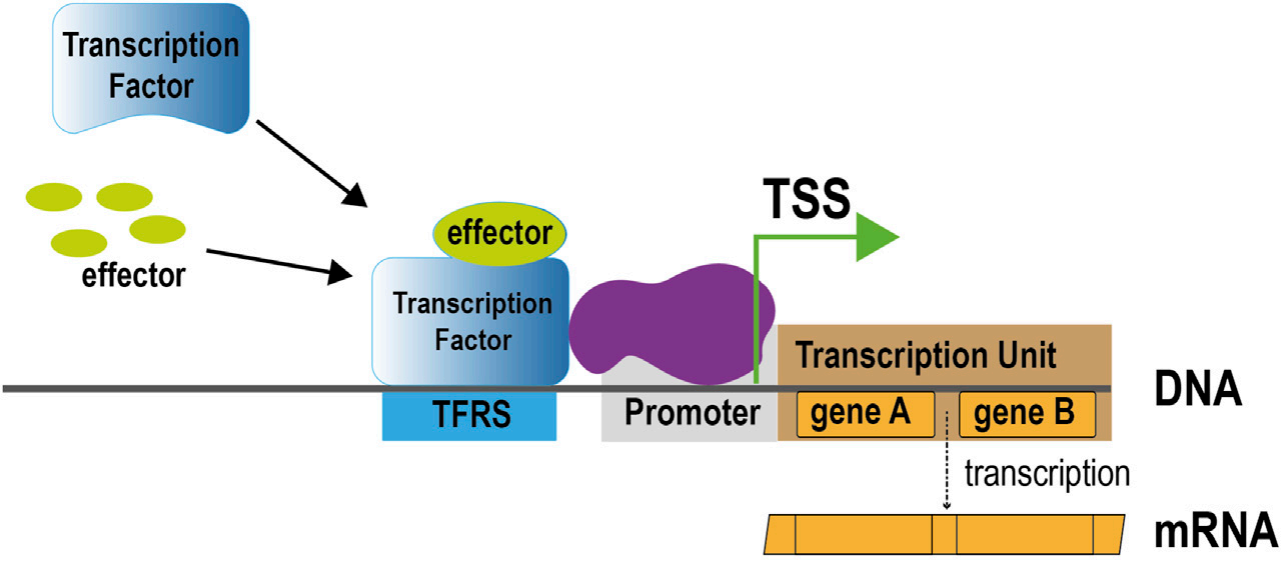
Main components of regulation of transcription initiation through allosterically regulated TFs. Upon binding of its effector, a TF changes conformation and alters its binding to regulatory sites in the DNA. Recruitment of RNA polymerase (shown in purple) promotes transcription of the transcription unit downstream of the regulatory region. TSS, transcription start site; TFRS, TF regulatory site.

Progress through the years of our biocuration efforts on regulation of transcription initiation and operon organization in *E. coli* K-12 has been periodically published, mostly in the special issues on databases in *Nucleic Acids Research* (Huerta et al., 1998; Gama-Castro et al., 2016; Santos-Zavaleta et al., 2019). The most common way to share progress has been in terms of the number of the main players of gene regulation, such as transcription factors (TFs), the operator DNA sequences to which TFs bind, called TF binding sites (TFBS), and TF regulatory sites (TFRSs) when there is evidence of their regulatory role in addition to binding, promoters, and other regulatory elements, including transcription start sites (TSSs) and transcription units (Figure 1). These elements include objects based on their interactions, such as regulatory interactions, that link TFs and their activating or repressing effects on the target genes, with detailed knowledge of the promoter involved for several of them, as well as operons and simple and complex regulons defined as the collection of target genes by a single TF, or by a group of TFs, respectively, (Mejía-Almonte et al., 2020). Together they define the transcriptional regulatory network of *E. coli*.

RegulonDB was originally organized within a conceptual framework that maintains a clean genomic picture following the classic definitions of the regulation of transcription initiation. However, these concepts required updating (Mejía-Almonte et al., 2020) to better represent the constant expansion of knowledge about transcriptional regulation, including the massive identification of regulatory elements made possible using high-resolution, genome-scale strategies (Seo et al., 2014; Yan et al., 2018; Ju et al., 2019).

### Regulation and Sensing Systems

In some well-studied systems, the *signal*, considered herein and in RegulonDB as the metabolite or physical change (e.g., temperature, pH, osmotic pressure, etc.) that initiates a flux of information that will affect the expression of one or a group of genes, has been identified and linked to the molecular mechanisms it triggers. In the well-known case of lactose metabolism (Pardee et al., 1958), in the absence of glucose, when the signal, lactose, reaches a certain concentration outside the cell, it is transported into the cytoplasm where it is isomerized to allolactose, a different but related molecule. This metabolite, which we call an *effector*, will bind LacI, the cognate TF, which will unbind from its operator sites. This unbinding of the repressor and the binding of CRP, the TF that responds to glucose, will induce the expression of genes involved in the metabolism of lactose.

In other more complex cases, the effector is known but the signal is less clear. For example, the TFs PdhR and IclR bind pyruvate, a central metabolite that can potentially change in abundance due to many different environmental triggers (Lorca et al., 2007; Anzai et al., 2020). To go from the conditions that provoke a genetic response to deciphering the molecular mechanisms that support such a response is not always straightforward. Experimentalists have to isolate the cause of changes in gene expression from additional cellular changes. For instance, changing from nitrogen-poor to nitrogen-rich sources will affect the growth rate, which by itself provokes additional changes in gene expression, making it harder to track the precise information flux from signal to effector of nitrogen-related TFs (Magasanik, 2000; Zimmer et al., 2000). Regulatory systems are also built based on responses to internal signals, and in fact, in several cases the effectors that bind to the TFs may have either an external origin as a metabolite transported into the cell or an internal origin resulting from an enzymatic activity inside the cell, or both. The distribution of internal and external signals in relation to local and global regulation has been previously analyzed (Martínez-Antonio et al., 2006).

### Information Transfer From Signal to TF

The information flux from the signal to the TF is achieved either by direct binding of the signal to the TF, or more frequently through a transduction process when the signal molecule is chemically transformed into the effector molecule which binds and alters the TF conformation. The current corpus of knowledge of *E. coli* sensory systems shows that by far the most frequent mechanism of this information transfer is achieved by means of allosteric interactions of specific metabolites that bind to the TF (Madan Babu and Teichmann, 2003).

**FIGURE 2:**
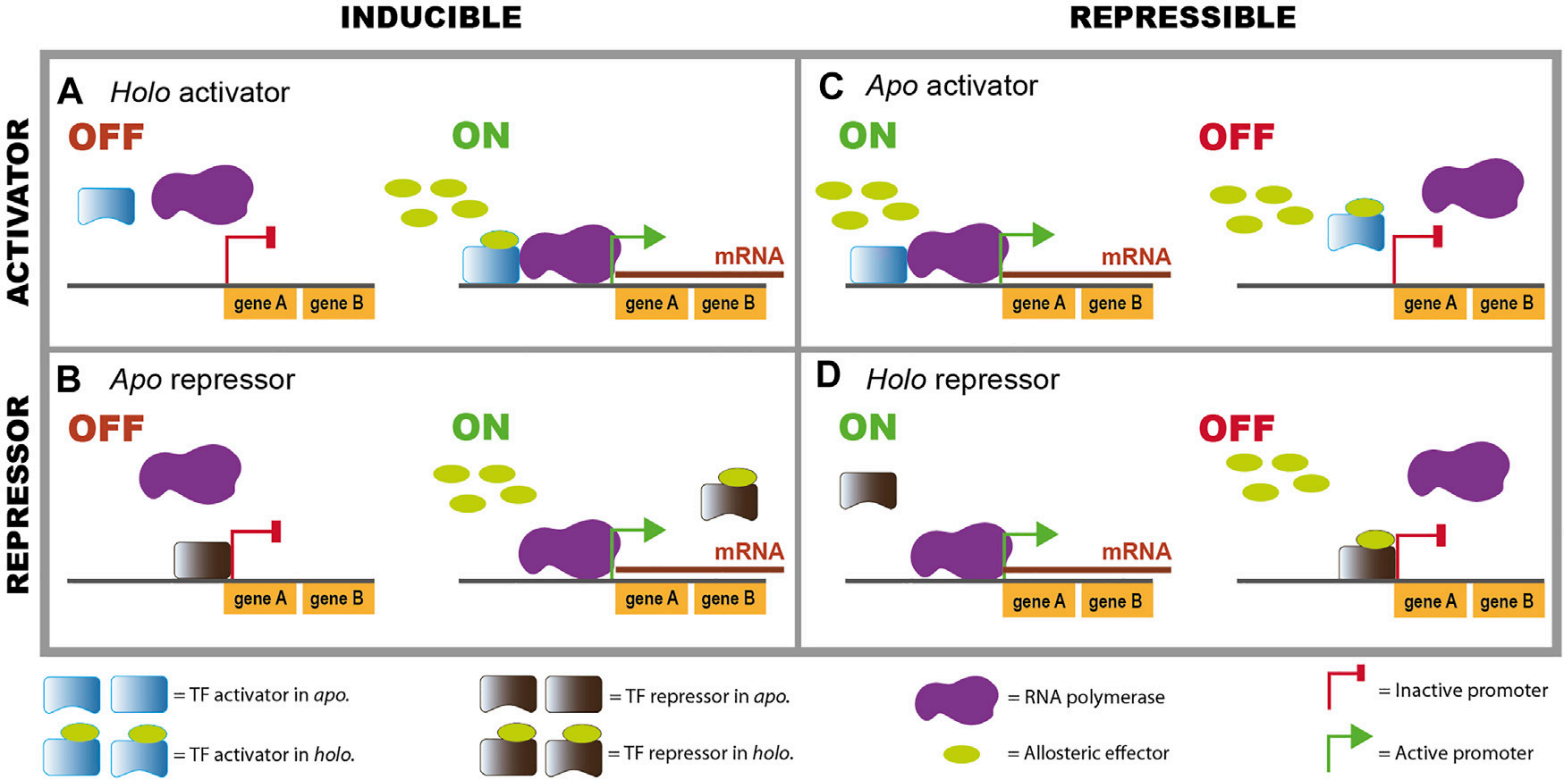
Combinations of regulatory effects of TFs over their regulated promoters, depending on their active conformation. Binding of an allosteric effector enhances or decreases DNA binding of TFs, producing an increase or decrease in transcription. All the different combinations exist in the compendia of *E. coli* TFs. Note that in some unusual cases TFs bind in both conformations. **(A)** *Holo* activator, **(B)** *Apo* repressor, **(C)** *Apo* activator, **(D)** *Holo* repressor.

For instance, for the lactose metabolism described above, LacI switches from its *apo* conformation as a free protein, which is the one that binds to TFRSs or operator sites, to its *holo* conformation as LacI-allolactose complex unbinding from its TFRSs. This explains the induction that lactose can exert on the regulated *lacXYZ* operon, via the unbinding of a repressor TF (Figure 2B). Another example is catabolite repression, a term used for the downregulation of several operons that encode enzymes for the utilization of alternative, nonpreferred carbon sources (Görke and Stülke, 2008). When glucose, which is largely considered the preferred carbon source in *E. coli*, is incorporated in the cell, it produces a decrease in the intracellular concentration of adenosine 3′,5′-cyclic monophosphate (cyclic AMP, or cAMP), the effector of the global CRP regulator. CRP is a transcriptional regulator of several operons involved in metabolism of different carbon sources; t binds to its TFRSs in a holo conformation as the complex CRP-cAMP. Thus, some operons are turned off in the presence of glucose (which leads to low cAMP levels), due to the unbinding of an activator that changes from its holo to its apo conformation (Figure 2A). Other TFs use mechanisms depicted in Figures 2B-D.

In summary, there are four possible combinations of activator or repressor and *apo* or *holo* conformations that bind to the operator sites (Figure 2). This is the general behavior although in some exceptional cases a TF can bind both in *apo* and in *holo*, and even upstream of the same promoter as illustrated by AraC (Schleif, 2010).

**FIGURE 3:**
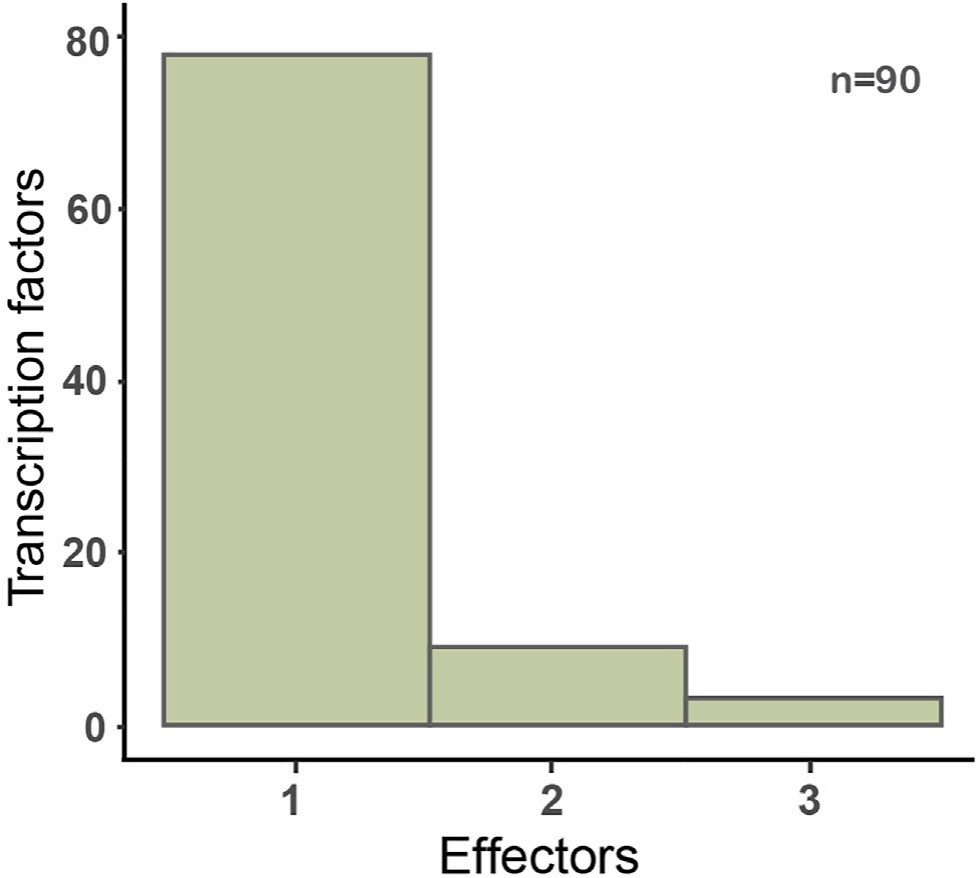
Distribution of the number of allosteric effectors for 90 TFs in *E. coli*. Only metabolite-binding TFs with experimental evidence of their interactions are included here. The full list is available on Supplementary Table S1.

The CRP regulon is an example of regulation by an allosteric metabolite, which is the most frequent mechanism of information flux from the signal to the TF. The first comprehensive compilation of the literature on the different conformations and metabolites that bind to TFs was reported by Balderas et al. (Balderas-Martínez et al., 2013). Since then, we have continued gathering this knowledge as it becomes available, so that currently of the 221 TFs with evidence of at least one regulatory interaction experimentally identified, we know the effector for 90 of them. Although by far current knowledge tells us that TFs respond to only one specific allosteric metabolite, in some cases TFs may sense more than two or even more different metabolites (Figure 3, Supplementary Table S1).

Some of those multiple effectors for a single TF, such as the metal-binding proteins ZntR and NikR, have been identified *in vitro*, but it is not fully certain that they have a physiological significance (Changela et al., 2003; Leitch et al., 2007). For instance, if the kinetics of the metal-TF complex only allows an interaction when metal concentrations are lethal for the bacterium, then the interaction is unlikely to have physiological significance. Another interesting case is the Lrp regulator whose main effector is leucine, but it has been shown that other amino acids, such as methionine and alanine can also bind allosterically to Lrp, but their physiological significance has not been well established (Hart and Blumenthal, 2011). It is important to mention that our TF-effector statistics reflect only those effectors which support regulatory interactions in *E.coli* K-12.

Less clear at this time is whether different conformations for the same TF can recognize different subsets of TFRSs, thus regulating different subsets of target genes. For instance, it was recently shown that, depending on the composition of the culture medium, different sets of genes are subject to regulation by the leucine-responsive regulator (Lrp) (Kroner et al., 2019). This is supported by other studies showing that binding of leucine to Lrp favors one specific Lrp conformation over others that coexist *in vivo*, increasing the DNA-binding affinity of Lrp to sites where it would not bind if leucine were not present (Chen et al., 2001; Chen and Calvo, 2002).

**FIGURE 4:**
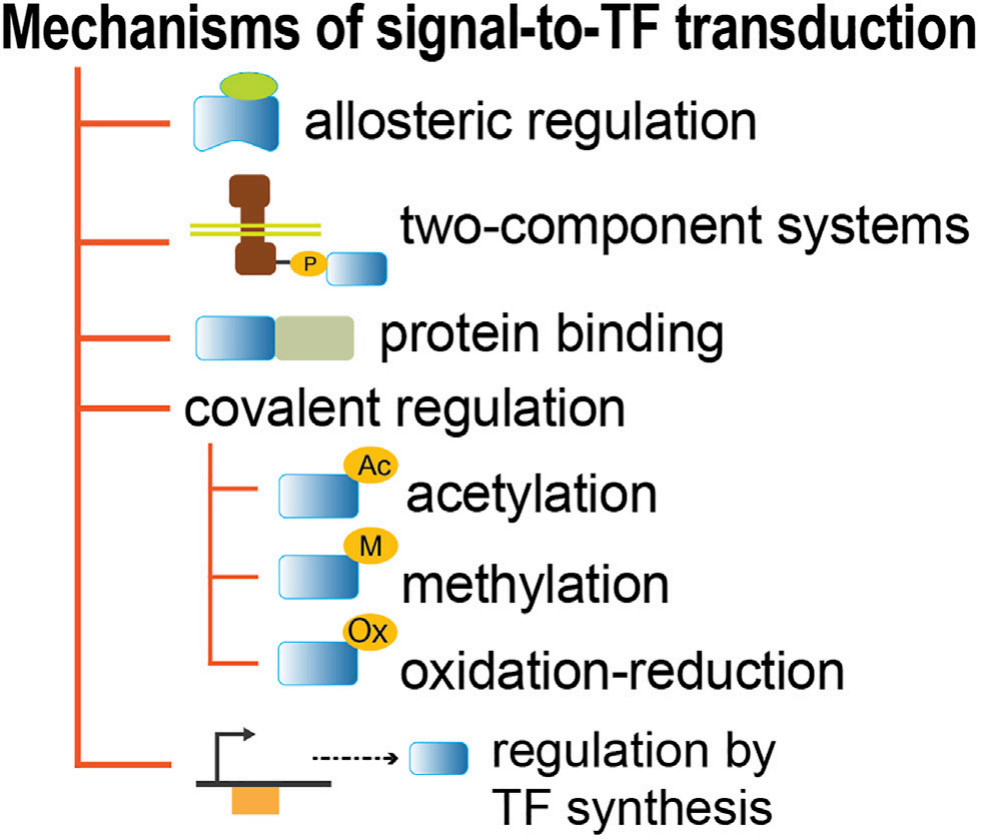
Mechanisms that connect extra- and intra-cellular environmental changes to TF activity.

The signal-to-TF information flux can also be executed by mechanisms different from allosteric binding to TFs (Figure 4, Supplementary Table S1). The two-component systems involve the covalent phosphorylation-dephosphorylation of the TF by the cognate histidine kinase sensor. In some cases, it has been shown that the kinase activity depends on the allosteric binding of an effector, as happens with ArcA (Jeon et al., 2001). Other TFs in bacteria rely on protein-protein interactions to modify the conformation capable of altering their DNA binding ability, such as toxin-antitoxin systems, as well as MalT (Schreiber et al., 2000; Joly et al., 2002; Mandrich et al., 2002; Schlegel et al., 2002), Mlc (Lee et al., 2000; Nam et al., 2001), and TorR, which are involved in responses to alkaline/acid stress in which the binding of TorI can affect recruitment of the RNA polymerase (Ansaldi et al., 2004). Other TFs, such as Ada, RcsB, and FNR, can be modulated by covalent modifications that include methylation, acetylation, and oxidation-reduction (Takahashi et al., 1988; Khoroshilova et al., 1997; Hu et al., 2013). The regulatory function of TFs can also be triggered by their own synthesis, without the presence of any effector metabolite. These usually have a slower response given the time needed to synthesize the full active protein in adequate concentrations. This is the case for some TFs like IHF, HNS, and other so-called nucleoid-associated proteins, whose changes in concentration are associated with changes of growth phase (Ali Azam et al., 1999; Azam and Ishihama, 1999).

Although it is not well understood why a particular mechanism is used in each signal-to-TF transduction process, the demand theory of gene regulation suggests a framework where the optimal solution is selected in evolution (Wall et al., 2004). Much remains unknown when searching for rational explanations, given the difficulty to test them. At the other extreme, TF orthologs may control different biological processes, as illustrated by CRP. Whereas this TF functions as the master regulator of catabolite repression in *E. coli*, its ortholog in *Pseudomonas* species regulates membrane-related functions (Milanesio et al., 2011).

Irrespective of the mechanisms for sensing, the signal induces a change in the conformation of the TF, and this change will either increase or decrease the concentration of active TF. This, in turn, will promote either binding or unbinding to its DNA target sites, exerting their regulatory effect on the corresponding regulated promoters. The sensing aspect ends by conveying the information originated by the signal to the genome. Here begins the genetic response encoded in the target genes of the regulated promoters. As a first approximation, the whole process can be described as a genetic sensory-response unit, or GENSOR unit for short.

### Genetic Sensory-Response Regulated Units

**FIGURE 5:**
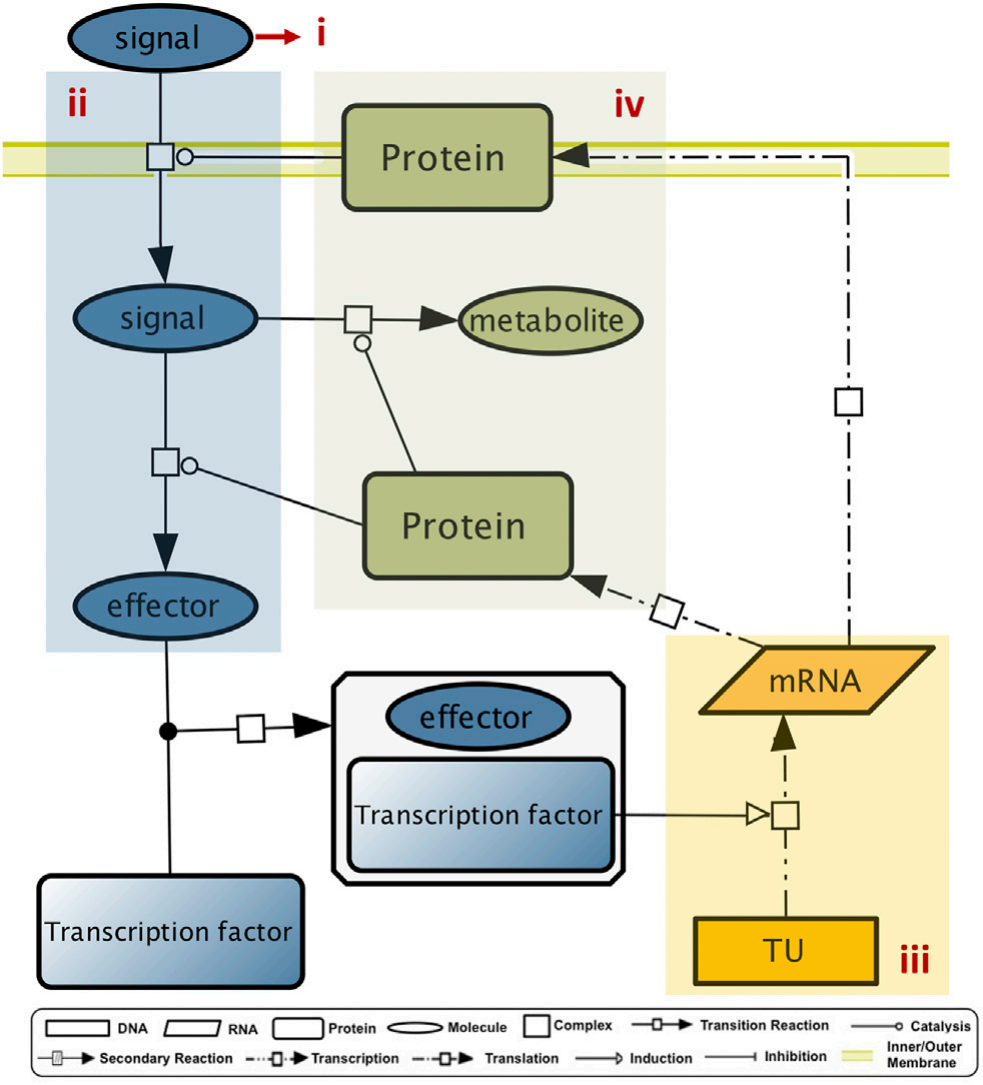
GENSOR unit components. A GENSOR units describes the flux of information from the signal (i) to its transformation into the TF effector (ii), to the genetic switch that the TF active conformation promotes (iii), to the coordinated metabolic response in which the regulated genes are involved (iv).

An elementary GENSOR unit includes the events from signal detection to the outset of a functional response, mediated by an individual TF. This process is summarized in four components: (Santos-Zavaleta et al., 2019) the signal, (Keseler et al., 2021) the conversion of signal to the effector, (Huerta et al., 1998) the set of genetic switches, and (Gama-Castro et al., 2016) the response (Figure 5, (Ledezma-Tejeida et al., 2017)).

Methodologically, the assembly of elementary GENSOR units centers around an individual TF and includes all the genes whose promoters are regulated by the TF, their mRNAs, their gene products, and the protein complexes they belong to. If the gene product is an enzyme, the reaction catalyzed, its substrates, and products are also included. From the information organized in RegulonDB and EcoCyc, we previously built GENSOR units for 189 TFs (these can be found in RegulonDB v10.9, grouped in different classes; http://regulondb.ccg.unam.mx/central_panel_menu/integrated_views_and_tools/gensor_unit_groups). The TF collection was updated for this work, (See Data Availability), and we now have 204 GENSOR units, for which an effector is described for 87 TFs. Each GENSOR unit is a multilevel network linking transcriptional regulation to metabolism by including the cellular components that are directly affected by the regulatory activity of a TF. They were used to predict metabolites allosterically regulating a TF, after the observation that 83% of TFs for which a binding molecule was known had it in their GENSOR unit (Ledezma-Tejeida et al., 2017). Further analyses focused on quantifying the functional homogeneity of the metabolic response mediated by a single TF. We have previously shown through several metrics that only ∼25% of TFs regulate one biological process, as opposed to two or more, and in 16% of them the gene products encode reactions that cannot be linked to classical pathways (Ledezma-Tejeida et al., 2019).

These are elementary GENSOR units, as they are limited to one TF. As discussed below under “Genomic processing of multiple signals in *E. coli*,” this is still a simplification of the complex architecture of sensing and genetic regulation exerted by TFs in orchestrating changes in gene expression. Nonetheless, they have been useful as a first multilevel integration, as discussed elsewhere (Ledezma-Tejeida et al., 2019). Furthermore, and as illustrated below, they are amenable to computational processing to identify if their response includes feedback as expected. GENSOR units must be periodically updated as new regulatory interactions are identified, particularly with the combination of high-throughput technologies for the identification of target sites for TFs in the genome and genome-wide expression profiles (Aquino et al., 2017; Santos-Zavaleta et al., 2019).

### Response to Signal Feedback

Signals trigger a change in the expression of one or more genes, and this response helps the cell to deal with what is implied by its presence in the environment. It is thus expected that the response includes feedback to the signal, for instance, the presence of carbon sources induces the expression of gene products used in their utilization, the presence of amino acids can repress their synthesis and save the energy needed in their synthesis. Furthermore, feedback to the signal should help TFs return to their previous state after transient changes. In order to explore this question, we reassembled GENSOR units by combining data from EcoCyc version 25.1 (Keseler et al., 2017) and RegulonDB version 10.9 (Santos-Zavaleta et al., 2019).

First, we focused on the simplest type of feedback: a metabolite signaling the TF is produced, transformed, or transported by an enzyme whose gene is directly regulated by the TF. We implemented a computational pipeline to automatically retrieve feedback from a GENSOR unit by identifying whether the effector molecule takes part in a reaction that is part of the GENSOR unit, excluding of course the effector-TF binding reaction (See the Material and Methods section, below). Of 90 TFs with information on their effectors, 87 had a GENSOR unit assembled. We found direct feedback in 71 GENSOR units, retrieving feedback computationally in 82% of the cases (for detailed results see Supplementary Table S2). Because we expected every sensory-response unit to have feedback, we analyzed in detail the remaining 16 GENSOR units where the feedback was not detected (Supplementary Table S3). We found four major reasons that explained why the feedback was not found:

1) The allosteric effector of the TF is not present in a metabolic reaction in the GENSOR unit but a compound either in the subclass or superclass (within the EcoCyc classification) of the effector is part of the GENSOR unit. For example, the effector of GalR D-galactose is not part of a metabolic reaction in the GENSOR unit, but a subclass of D-galactose, α-D-galactopyranose, is found in a reaction that is part of the GENSOR unit. Similar cases were found for AraC, ExuR, FadR, GalR, GalS, RbsR, and TreR. In Supplementary Table S3 we show the allosteric effectors and the metabolites occurring in other reactions in the GENSOR unit. For these cases, we consider the feedback to be supported by evidence even if it was not found computationally.
2) The second case is that of TFs that must be analyzed in connection with additional TFs in which the feedback is not automatically detected unless reactions from a second regulator or regulated TF are considered. This is the case for AllS, Cbl, and RhaR. Allantoin is the effector of both AllS and AllR, and AllR regulates *allS* as well as *allB*. Since the enzyme encoded by allB catalyzes the first step in the assimilation of allantoin, the missing feedback of AllS is recovered when considering that it is regulated by AllR. The allosteric effector of Cbl is adenosine 5′-phosphosulfate, which is synthetized by the enzyme sulfate adenylyltransferase, encoded by *cysD* and *cysN*; both genes are regulated by CysB, which in turn is regulated by Cbl. Thus, these CysB-regulated reactions have to be considered to identify the feedback of Cbl. For RhaR, the allosteric effector is α-L-rhamnopyranose, a subclass of L-rhamnose, whose transport is mediated by RhaT, transcriptionally regulated by RhaS, which in turn is regulated by RhaR. Thus, the biological feedback is clear but is mediated by an additional TF.
3) We found two GENSOR units in which the feedback was not automatically identified for TFs that have an enzymatic function, BirA and DnaA. Up to now these additional activities of TFs have not been included in the GENSOR units. BirA is a DNA-binding transcriptional repressor and a biotin-[acetyl-CoA-carboxylase] ligase, and its allosteric effector is biotinyl-5′-adenylate. BirA negatively regulates BioB, which synthesizes biotin, and biotin is used by BirA to produce biotinyl-5′-adenylate. When biotin is in excess, it is transformed to biotinyl-5′-adenylate by BirA, and BirA-biotinyl-5′-adenylate negatively regulates *bioBFCD* involved in synthesis of biotin (Sirithanakorn and Cronan, 2021).Then the feedback is evidenced when this additional activity of BirA is taken into consideration. On the other hand, DnaA is a transcriptional dual regulator and a chromosomal replication initiator protein with ATPase activity. The allosteric effector of DnaA is ATP. When DnaA interacts with two homodimers of Had, the DnaA-bound ATP is hydrolyzed (Su’etsugu et al., 2005). Hence, once the hydrolysis reaction is included, the feedback becomes evident.
4) Finally, in other cases identifying the feedback is hard because of the limited knowledge for the components of the GENSOR units, and no clear conclusion can be made. We consider this to be the case for MarR, PurR, PyrR, and ComR. In the case of the MarR GENSOR unit, no enzyme has been reported in the genome to produce, consume, or transport salicylate, which is the allosteric effector. PurR regulates enzymes whose reactions are involved in IMP, a precursor of hypoxanthine, its allosteric effector. ComR regulates BhsA, a multiple stress resistance outer membrane protein that appears to reduce the outer membrane permeability to copper (Mermod et al., 2012), its allosteric effector. Once this process is characterized and a reaction of inhibition of copper uptake is annotated, it will be possible to identify automatically the feedback for ComR. Finally, the only target gene identified to date for PyrR (whose effector is pyruvate) is YhjX, an ABC transporter of unknown function. It has been reported that YhjX forms hetero-oligomers with YjiY, a pyruvate transporter; however, it is not yet known if YhjX affects pyruvate transport by YjiY (Behr et al., 2014).

These detailed analyses show that all GENSOR units have evidence of feedback, except for four cases which lack sufficient data (Supplementary Table S2). The computational program used to identify feedback is publicly available (https://github.com/PGC-CCG/Feedback-in-GUs) and can be used to search for a metabolite in any collection of reactions. The detailed curation work that expanded the feedback discovery illustrates both the complexities in the biology of signaling processing and the concomitant difficulties in the adequate representation of this knowledge in databases. Our analysis here was limited to TFs with allosteric effectors, and a similar analysis should be done with all other mechanisms depicted in Figure 4.

### Biosensors Based on TFs

*E. coli,* in addition to being a model for the study of microbial physiology, has been widely used as a “chassis” for synthesis of valuable compounds through metabolic engineering. The integration of genetic regulation knowledge during metabolic engineering design is mandatory to improve the titer, yield, and productivity to afford the processes that are economically feasible. In particular, TFs modulated by signal metabolites are considered valuable tools for metabolic pathway engineering, since they can be used for monitoring or to control metabolic fluxes in the production of biofuels, organic acids, polymer precursors, and drugs, among others (Li et al., 2020).

Whole-cell TF-based biosensors have been constructed with a TF sensing a chemical compound modulating a reporter gene (Fernandez-López et al., 2015). These systems have been employed for high-throughput library screening to select new bacterial strains with desired metabolic characteristics (Kaczmarek and Prather, 2021). Similarly, TF-based biosensors can be used to monitor organic and inorganic pollutants in industrial sewage or in the environment. The high sensitivity of TFs enables the detection of pollutants in concentrations below the limit of some analytic techniques, quickly and cheaply. Examples include cadmium and mercury sensing by CadR (Tao et al., 2013). Manipulation of transcriptional regulation to improve the yield of an engineered system can be achieved in several ways, for example, by constructing hybrid promoters to reduce the concentration of toxic intermediaries in synthetic pathways (Zhang et al., 2012) or by increasing the flexibility of the engineered process. A successful case of this strategy was reported by Zhang et al. (Zhang et al., 2012), in which the yield and titer of biodiesel production by a bacterial strain were improved. The strain was previously engineered to overexpress ethanol (precursor), fatty acids (precursors) and fatty acid ethyl esters (biodiesel) biosynthesis pathways. In that report, a synthetic promoter that includes a 17-bp FadR-binding DNA sequence was cloned upstream of *adhB* and *pdc* (encoding the enzymes for the ethanol biosynthesis), resulting in their repression by FadR. As a consequence, only when acyl-CoAs are present, the ethanol biosynthetic pathway is transcriptionally derepressed and biodiesel synthesis is carried out, thus avoiding high concentrations of ethanol in the cell, improving the stability of the strain (Zhang et al., 2012).

Supplementary Table S1 in the supplementary material contains the list of effectors for the 90 TFs with currently characterized noncovalent effectors. We consider this repertoire of TFs involved in naturally sensing different metabolites as a valuable resource for bioengineering purposes. Moreover, GENSOR units could be a reference point from which to search key compounds of biosynthetic pathways. If the signal, effector, or an intermediate of a GENSOR unit is present in the process of interest, the transcriptional regulator can be integrated into the model for monitoring or directing metabolic fluxes.

The implementation of TFs as biosensors has shown to be useful in stabilizing the yield for the production of several compounds, however, to date this biotechnological application is limited to well characterized TF-effector regulatory interactions. Furthermore, the knowledge of transcriptional regulatory elements in *E. coli* and other bacteria is rapidly expanding with technologies to identify the binding of TFs in the whole genome (Santos-Zavaleta et al., 2018), increasing for instance, the number of TFs with known binding sites in *E.coli* to 189 TFs (Gao et al., 2021). Future progress should expand to eventual completion of the TF-gene regulatory repertoire of interactions, enabling the definition of complete GENSOR units; this information shall improve the use of *E.coli* as a chassis in synthetic biology. Also, insights into molecular mechanisms governing allosteric interaction and regulatory effects could be employed to engineer allosteric TFs to sense new compounds, which could allow the use of TF-based biosensors in a wider range of biosynthetic processes (Tao et al., 2013; Taylor et al., 2016).

Most applications have exploited and implemented one signal-sensing system. As mentioned before, the GENSOR units we have discussed are constructed around a single TF. We know, however, that in addition to the predominantly one-to-one relationship between signals and TFs (Figure 2), transcriptional regulation offers an additional layer of integration at the level of promoters.

### Genomic Processing of Multiple Signals in *E. coli*

Transcriptional regulators bind around one or multiple promoter regions to control the expression of the downstream gene or collection of genes organized in polycistronic units in bacteria. The GENSOR units we have discussed were built using the group of genes that have a binding site for a particular TF in their upstream promoter region, that is, a simple regulon. However, the architecture of transcription can be rather complex with multiple nearby promoters upstream of genes and multiple binding sites for different TFs (Mejía-Almonte et al., 2020). Given the finite number of TFs and promoters in a genome, different productive approaches have modeled gene regulation from a combinatorial perspective, either with grammatical models to describe the multiple combinations of sites for different TFs in a promoter region (Collado-Vides, 1992), in quantitative thermodynamic models (Bintu et al., 2005a; Bintu et al., 2005b), or in combinatorial logic modules of gates of regulation (Buchler et al., 2003).

**FIGURE 6:**
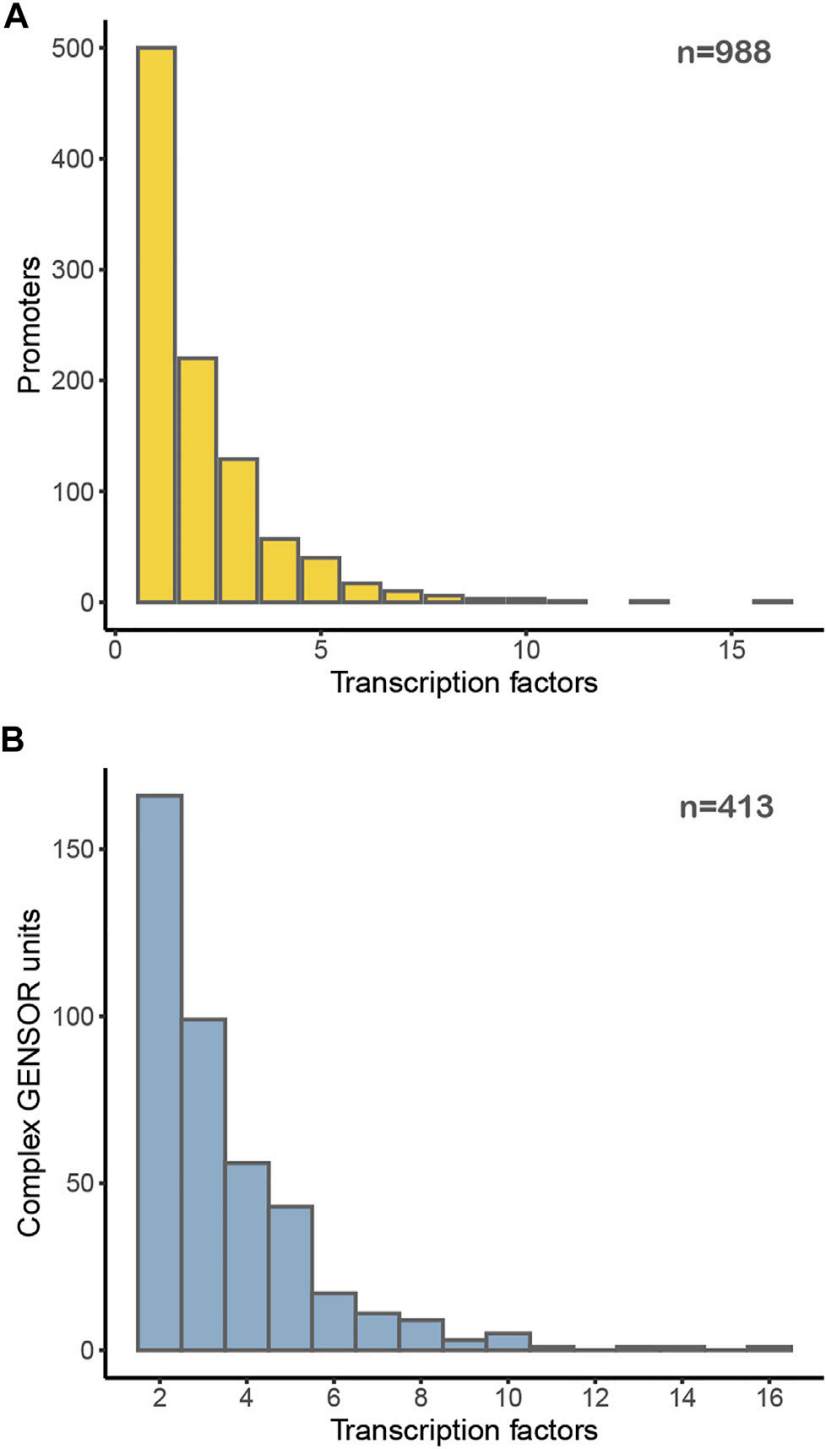
Distribution of TFs regulating **(A)** each promoter in *E. coli* or **(B)** participating in a complex GENSOR unit.

We can think of this genomic organization as supporting the collection of decisions genetically encoded in the genome. An interesting question is what is the number of signals that the genetically encoded collection of decisions integrates. In the first layer, that of the number of effectors binding to TFs (Figure 3), it is hard to say it conveys integration, since in the reduced number of cases of multiple effectors, it is not clear if multiple binding happens under the same growing conditions. Thus, we consider that, most likely, there is no major integration of signals in decision processes at this level. Figure 6A, however, shows that approximately 50% of promoters are subject to regulation by multiple TFs, with a distribution of up to ∼10 different TFs affecting the same promoter. Although we cannot say that in all these cases the different TFs simultaneously affect promoters or if they bind separately under different conditions, in a good number of well-studied cases it has been shown that multiple TFs work together in affecting promoter activity. Projects requiring quantitative approaches could benefit from the combinatorial repertoires in the approaches we have mentioned before, those of the Terry Hwa (Buchler et al., 2003) and Rob Phillips (Bintu et al., 2005a; Bintu et al., 2005b) laboratories. Figure 6B shows the number of complex GENSOR units, defined based on complex regulons and their jointly regulated target genes. Genetic decisions at the level of transcription initiation can integrate the simultaneous occurrence of multiple signals, as illustrated by carbon source decisions involving the absence of glucose mediated by the catabolite repressor CRP and the presence of another sugar (e.g., lactose, maltose, or arabinose with LacI, MalT, or AraC, respectively), to mention a few well-known cases.

We consider complex GENSOR units to better reflect the activity of decision-making in *E. coli* than elementary GENSOR units, as they are derived from the organization of TFs regulating promoters in the genome. This is further supported by the increased correlation with co-expression of complex GENSOR units at a level similar to that of transcription units (see Figure 4 in (Ledezma-Tejeida et al., 2019)).

Despite these warnings, Figure 6B shows our best approximation to the distribution of the number of different signals that regulatory TF-based decisions are integrated by these sensing processes.

## Discussion

The transcriptional network of the *E. coli* genome is estimated to involve ∼300 TFs (Pérez-Rueda and Collado-Vides, 2000; Madan Babu and Teichmann, 2003), of which we have experimental evidence for 221 TFs and evidence of allosteric interactions for 90 of them (40%). Recalling that two-component systems also have an allosteric component, this number could increase to 118 (53%). However, sequence-based computational predictions estimate that 75% of the complete set of TFs bind small molecules (Madan Babu and Teichmann, 2003), a higher fraction than what is currently experimentally supported.

It is remarkable to note the few number of allosteric effectors that bind overall to TFs, as shown in Figure 4, with 1.16 effectors per TF on average. It is possible that this number is greatly underestimating the TF-metabolite interactions that happen *in vivo*, since, as already mentioned, these numbers are limited to the effectors for which there is evidence of a regulatory interaction. A recent *in vivo* systematic analysis combining transcriptomics and metabolomics identified new effectors for 30 TFs, in many cases increasing the number of known effectors for a given TF (Lempp et al., 2019). Although high-throughput analysis inherently identify false positives and the authors of the study acknowledge that a disadvantage of their method is the false identification of metabolites with similar dynamics to the true effectors, studies in enzyme-metabolite interactions have also identified a large number of potential ligands *in vivo* (Piazza et al., 2018). The compendia of TF-metabolite interactions presented here is backed up by *in vitro* experimental evidence, it is likely that the list will grow as more systematic TF-metabolite identification studies appear, and the challenge will shift to identifying those that are functionally relevant. Alternatively, TFs might have been selected to have a reduced number of allosteric interactions favoring the one-to-one mediated information flux from signals to promoters as opposed to enzymes (Huang et al., 2011).

As we have discussed, this implies that the integration of multiple signals happens in bacteria, predominantly, via the binding of multiple TFs governing the level of individual promoters. Transcriptional regulation is also governed by TFs that change their conformation via different mechanisms, as schematized in Figure 4. The mechanisms can be rather complex, like the multiple cascades of enzymatic transformation in two-component systems (van Heeswijk et al., 2013; Groisman, 2016). Also as mentioned, other TFs seem to have no multiplicity of conformations but are regulated directly by their synthesis, like IHF. We are aware that this variability supports a diverse dynamic in the signaling processes used in *E. coli*.

We have argued that sensing is an essential aspect of the larger context of gene regulation when considering those cases that evolution has generated to enable the cell to address changes in the environment. The genetic sensory response units, or GENSOR units, we previously assembled fit perfectly with this perspective. GENSOR units are multilayer in the sense of grouping transport, signaling, gene regulation, the regulated gene products, and either enzymes or any other product, as well as the reactions they encode, and the metabolites involved. They are preprocessed constructions based on the set of regulated genes that comprise the regulon of individual TFs. Their construction offers the highest level of integration currently available of major groups of co-regulated genes and their gene products. We have generated short summaries for several GENSOR units, describing the function of the regulated genes.

As mentioned already, elementary GENSOR units have enabled evaluations of the partial homogeneity of the functional putative response of regulons. We say “putative,” assuming all genes of a regulon are co-expressed, which we know is not the case. In that sense, complex GENSOR units define groups of genes with a much higher co-expression correlation, as mentioned before. Nonetheless, there is room for further work both experimentally as well as in the bio-curation of the regulatory modules or phrases governing complex GENSOR units to gather evidence and distinguish promoters subject to distinct regulation under different conditions, from promoters subject to simultaneous regulation by multiple TFs.

Elementary GENSOR units were used to confirm the hypothesis that feedback is inherent to regulatory circuits devoted to address changes in the environment. As mentioned in methods, we updated the holo TF conformations, so that now they are consistently using the main name of the bound effector, enabling computing with this knowledge. These changes will be uploaded in these databases, and the list of all allosteric effectors is included in the supplementary material. As mentioned, we automatically identified feedback in 82% of the cases and manually curated the remaining cases, identifying the presence of feedback in all of those with available information. Although this analysis was limited to allosteric-mediated regulation, to the extent that all TFs are part of the sensing capability of the cell, we believe that feedback is equally expected irrespective of the signaling mechanism. A different scenario could be imagined for gene regulation in, for instance, developmental programs, where feedback to the signal might not be logically required.

The accumulated knowledge of sensing and regulated circuits has provided the basis to develop many biotechnological applications, exploiting also the rich promoter architectures, as they are based on the identification and rational manipulation of those circuits or parts of them (Zhang et al., 2012; Tao et al., 2013; Taylor et al., 2016; Graham et al., 2020; Li et al., 2020). Genome identification of whole-genome transcriptional regulatory networks may have a concomitant impact in a new generation of biotechnological applications exploiting more complex metabolic and regulatory circuits.

## Materials and Methods

### GENSOR Unit Assembly

GENSOR units from the regulatory network in the format “TF-gene” available in RegulonDB v10.9 were assembled using the pipeline described in Ledezma-Tejeida, NAR, 2019, using custom Perl scripts available at (https://github.com/PGC-CCG/Feedback-in-GUs (Gensor option in the Gensor-Unit pipeline), originally published in https://github.com/dledezma/gensor_units.

### Curation of TF Conformations

In order to computationally identify the feedback, we revised the annotated active conformations of TFs to precisely name *holo* TF conformations using the main name of the bound effector, and not a synonym or short name as has been done before. These changes will be uploaded in these databases, and the list of all allosteric effectors is included in the supplementary material*.*

### Identification of Feedback in GUs

The feedback loops were identified directly from the GENSOR units assembled in the previous step. We then used a custom script (feedback option of the Gensor unit pipeline available at (https://github.com/PGC-CCG/Feedback-in-GUs) that was incorporated as input for the curated active and inactive TF conformations from RegulonDB v10.9 to define the metabolic effector or effectors that interact with each TF. Each GENSOR unit was then scanned for the presence of the effector or effectors as reactants and/or products in its reactions. Feedback was assigned to a TF if one or more of its effectors were found in its regulated metabolic reactions. For some TFs, the specific stereoisomers of the effector molecules were not found in the reactions in their exact form, but a lower- or upper-class compound were. In these cases, the program suggests possible matches in feedback, as there could be a lack of knowledge of their specific spatial conformation. These cases, however, must be curated manually.

The manual curation was carried out by analyzing the compounds in non-allosteric reactions from the GENSOR unit. The reactions in which the effector is present were retrieved, and then the regulation over the genes encoding the corresponding enzymes was analyzed.

## Data Availability

Publicly available datasets were analyzed in this study. This data can be found here: https://github.com/PGC-CCG/Feedback-in-GUs.
